# Single-port thoracoscopic removal of an azygos vein aneurysm: a case report and literature review

**DOI:** 10.1186/s13019-023-02143-2

**Published:** 2023-01-16

**Authors:** Yi Yao, Qiuxia Hu, Xiaoyang Xie, Caiyang Liu, Yu Lei, Xiaoliang Li, Yi Wang, Gaohua Liu, Yanhui Yang, Lei Luo, Ji Li

**Affiliations:** 1 Department of Cardiothoracic Surgery, The First People’s Hospital of Neijiang, No. 1866, West Section of Hanan Avenue, Shizhong District, Neijiang, 641000 Sichuan China; 2 Department of Obstetrics and Gynecology, The First People’s Hospital of Neijiang, No. 1866, West Section of Hanan Avenue, Shizhong District, Neijiang, 641000 Sichuan China

**Keywords:** Azygos vein aneurysm, Hemangioma of the azygos vein arch, Single-port thoracoscopic surgery, Three-dimensional reconstruction, Case report

## Abstract

**Background:**

Azygos vein aneurysms (AVAs) are extremely rare. The majority of patients have no obvious clinical symptoms, so they are found by physical examination or by chance. There is limited clinical treatment experience that can be referred to, and there are no clear guidelines or research evidence standardizing the surgical and interventional therapy. Here, we report a patient with idiopathic AVA whose three-dimensional reconstruction of the tumor was completed before surgery. On the basis of three-dimensional reconstruction, single-port thoracoscopic resection of the AVA was successfully completed and reported for the first time. The previously reported cases are summarized to provide guidance for the diagnosis and treatment of patients with AVAs.

**Case presentation:**

A 56-year-old man was transferred to our hospital due to “dysphagia”. The diagnosis of AVA was made after enhanced computed tomography, gastroscopy, fiberoptic bronchoscopy, and three-dimensional reconstruction. Congenital weakness or degenerative changes causes the vein walls to be extremely thin that the AVA had the risk of ruptur. Furthermore, the patient had symptoms of dysphagia, he received single-port thoracoscopic surgery. After the operation, his dysphagia disappeared. The postoperative pathology confirmed hemangioma. The patient was discharged 3 days after surgery without any complications.

**Conclusions:**

AVAs are rare. Preoperative three-dimensional reconstruction can greatly help surgeons clarify the disease diagnosis, formulate the surgical plan, avoid damage to the surrounding vital organs, and reduce intraoperative bleeding. Thoracoscopic surgery to remove AVAs is difficult and has a high risk of bleeding, while more minimally invasive single-port thoracoscopic surgery is also safe and effective for the treatment of AVAs.

## Background

Azygos vein aneurysms (AVAs) are rare [1]. In radiologic studies, the normal diameter of the AV is defifined as no more than 1 cm [2]. Underlying causes of AVA formation that have been proposed include cardiac decompensation [3, 4], portal hypertension [4, 5], pregnancy [3], and compression of the SVC due to neoplasms [3, 5]. It may occur rupture, thromboembolism, mediastinal mass effects, and pulmonary artery hypertension with progressive gradual enlargement of the AVA [3]. Because most patients are asymptomatic, the diagnosis is mostly an incidental finding. The optimal management of AVAs remains uncertain, and there is no clear distinction of criteria indicating conservative and surgical or interventional therapy. Usually, surgical resection is required in cases of compression of adjacent structures, appearance of clinical symptoms, azygos vein (AV) thrombus formation in patients with oral anticoagulation, contraindication to oral anticoagulation, pulmonary embolism, and considerable increase in the diameter of an AVA [1]. Due to the high difficulty of thoracoscopic surgical resection and the risk of embolism migration. Most patients underwent thoracotomy to remove AVAs, and only a few cases reported successful thoracoscopic resection [1]. There is no report of single-port thoracoscopic resection of an AVA. On the basis of three-dimensional reconstruction, we report a case of single-port thoracoscopic-based management of an idiopathic AVA with symptoms of dysphagia. At the same time, we review the symptoms, diagnosis, and treatment of AVAs in the published literature.

## Case report

**Fig. 1:**
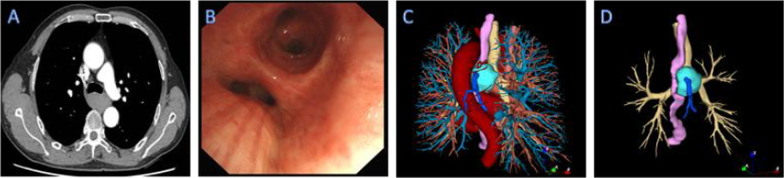
**A** Chest contrast-enhanced CT revealed that the mass located at the tracheal bifurcation. **B** Bronchoscopy revealed external pressure stenosis at the bronchial origin of the left lower lobe. **C** Three-dimensional reconstruction showed that the mass is a hemangioma originating from the azygos vein arch. There were two arteries from the aorta running among the tumor body, the esophagus and the trachea. **D** The tumor body obviously extruded the esophagus and trachea

A 56-year-old man complained of dysphagia. He was referred to our hospital without special treatment because of a mediastinal mass on chest computed tomography (CT). The patient had no relevant medical history. He denied any falls or other trauma. Body temperature was 37.0 °C, heart rate was 78 beats/min, blood pressure was 127/74 mmHg, and respiratory rate was 20 breaths/min. Breath sounds were normal. No moist or dry rales. No heart murmurs or muffled heart tones. Contrast-enhanced CT revealed a mediastinal mass (4.2 × 3.7 × 2.6 cm) located at the tracheal bifurcation. It has smooth borders with no obvious nodules. No other aneurysmal malformations were detected. The esophagus was obviously compressed on the corresponding plane. At the same time, contrast-enhanced CT suggests unremarkable enhancement in the arterial phase. It showed delayed enhancement with a CT value of approximately 118 HU in the venous phase (Fig. 1A). Bronchoscopy revealed external pressure stenosis at the bronchial origin of the left lower lobe (Fig. 1B). Gastroscopy showed no obvious abnormalities. The initial diagnosis was hemangioma or bronchial cyst. Three-dimensional reconstruction showed that the mass was connected to the AV and superior vena cava. The mass body obviously extruded into the esophagus and trachea. There were two arteries from the aorta running among the mass body, the esophagus and the trachea (Fig. 1C, D). These findings revealed that the mass is considered to be initial diagnosed as a hemangioma originating from the azygos vein arch. Surgical resection should take care with the aortic branch behind the AVA to prevent uncontrollable bleed. At the same time, we need to protect the vagus nerve and the thoracic duct. Avoid damaging them to prevent gastrointestinal symptoms and chylothorax.

**Fig. 2:**
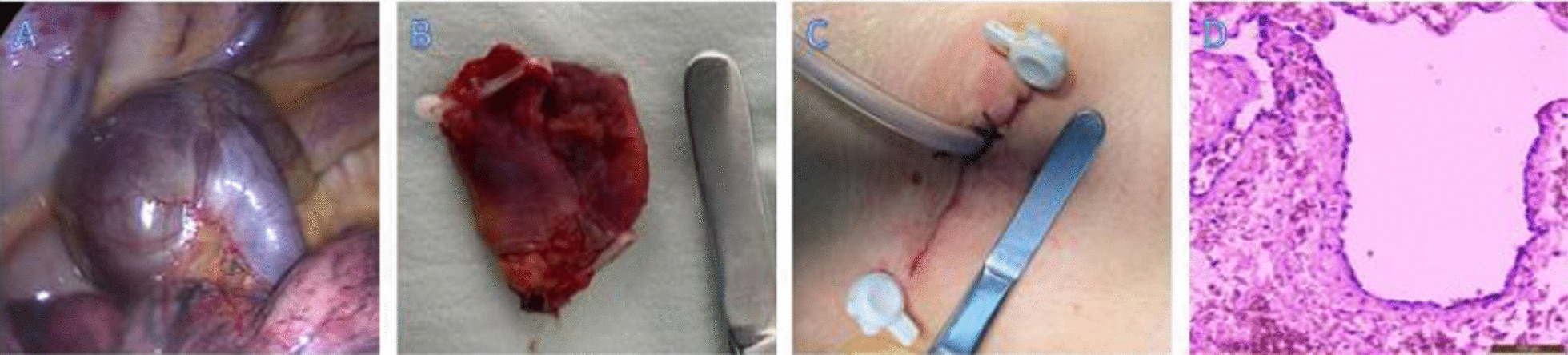
**A** exploration revealed that the AVA was located in the azygos vein arch. **B** the venous aneurysm was completely removed. **C** A 16F drainage tube was placed in the surgical incision after single-port thoracoscopic surgery. **D** Histopathology showed thinning of the AV wall and loss of the smooth muscle layer of the vascular wall

Dysphagia caused by AVA extruded the esophagus and the left bronchus. Interventional treatment may can’t cure dysphagia symptom. In addition, interventional treatment may not prevent future migration and embolization of the thrombus. So, we chose single-port video-assisted thoracoscopic surgery (VATS) to respect the AVA under double-lumen tracheal intubation 3 days after hospitalization. The patient was in the left lateral decubitus position during surgery. The intraoperative exploration results were consistent with the three-dimensional reconstruction. The AVA was located in the azygos vein arch, and there was no abnormal pulmonary artery or pulmonary vein connected with it (Fig. 2A). The AVA obviously extruded into the esophagus and the left bronchus, and the AVA tightly adhered to the esophageal wall. We used an electrocoagulation hook and an ultrasonic scalpel to open the mediastinal pleura at the junction of the AVA and the superior vena cava, denuded the AV, and cut it off with an ultrasonic scalpel after hemolock clip was clamped. Then, the stump end was pulled to separate the tumor from the left main bronchus and esophagus wall. The ultrasonic scalpel and electrocoagulation hook were used to dissociate the surrounding tissue of the AVA. The aortic branches behind the tumor were carefully separated, the ultrasonic scalpel was cut off, and the venous aneurysm was completely removed (Fig. 2B). After the operation, a 16-F drainage tube was placed in the surgical incision (Fig. 2C). The operation lasted approximately 60 min, and intraoperative blood loss was 20 ml. The patient recovered well after surgery. All symptoms of dysphagia disappeared after the operation, and the patient was discharged 3 days after the operation. Histopathology showed that the venous layer to be affected is the media. Thinning of the AV wall and loss of the smooth muscle layer of the vascular wall. No malignant cells were seen. So it was considered an idiopathic AVA (Fig. 2D). At the 1-month follow-up, CT showed no recurrence.

## Discussion

AVA is a rare disease. AVA is generally defined as a diameter of the AV exceeding 2.5 times the normal diameter. According to its morphology, it is currently divided into fusiform AVAs, with the overall expansion of the AV, and saccular AVAs, with local expansion of the azygos wall [3]. According to the etiology, it can be divided into (1) idiopathic AVA, (2) acquired AVA, and (3) traumatic AVA [1]. As far as we know, a total of 73 patients with AVAs have been reported in the available literature, who are summarized in Table 1 [3–63]. Among them, there were 29 males and 41 females. Their ages ranged from 3 months to 86 years (mean, 56.2 years), and their tumor diameters ranged from 1.2 to 15 cm (mean, 4.4 cm). There were 14 cases of fusiform AVAs and 32 cases of saccular AVAs. There were 54 cases of idiopathic AVAs and 6 cases of acquired AVAs. However, 3 patients' AVAs was accidentally discovered from trauma, and none were proven to be caused by trauma. Summarizing the previously reported cases, most of the patients were asymptomatic or had their AVA discovered incidentally on physical examination for other reasons. Among the common symptoms in patients, 13 patients had chest pain [12–15, 17, 18, 20, 22, 26, 35, 44, 48, 60], 10 patients had chest tightness and even dyspnea [3, 6, 19–21, 23, 25, 45], 4 patients had cough [3, 9, 43, 53], 3 patients had symptoms of dysphagia [19, 43, 59], and 3 patients' AVAs were accidentally discovered in trauma [3, 11]. Contrast-enhanced CT and magnetic resonance imaging (MRI) are the main noninvasive methods for the diagnosis of AVA. CT was completed in 72 patients of all reported 74 patients, and 22 patients completed MRI. Further completion of gastroscopy and fiberoptic bronchoscopy is an important supplement to CT and MRI when patients have symptoms such as dysphagia and dyspnea [6, 60]. However, poor tumor enhancement or intratumoral thrombus formation in the AVA may lead to misdiagnosis of AVA as a solid tumor or mediastinal lymphadenopathy [24]. When a mediastinal mass does not exclude the diagnosis of azygosmosis, needle biopsy is not recommended because of the risk of massive bleeding [25, 26]. In the present case, the three-dimensional reconstruction of the patient showed that the tumor was located in the AV, and the tumor was connected with the superior vena cava and the AV, which confirmed the diagnosis of AVA. At the same time, the three-dimensional reconstruction showed the surrounding tissues of the tumor. The tumor was closely related to the esophagus and airway. There were two arteries from the aorta running among the tumor body, the esophagus and trachea. During the operation, it was found that the tumor and esophagus were tightly adhered. When separating the posterior part of the tumor, we predict the location of arterial branches in advance. A combination of an ultrasonic scalpel and electrocoagulation hook was used to carefully separate the area of the aortic branch to avoid the risk of bleeding. Therefore, preoperative three-dimensional reconstruction plays an important role in the diagnosis of AVAs and the formulation of surgical plans [6, 8, 16, 33].

**Table 1:** Reported cases of Azygos Vein Aneurysm

Study	Age (y)	Sex	Symptom	Origin	AVA Type	Measuring (cm)	Diagnosis	Associated diseases	Treatment	References
Ko	33	M	Chest tightness	Idiopathic AVA	Saccular	6.5 × 6.0	CXR, CT, MRI	None	Surgical resection	[3]
Ko	72	F	Chest tightness	Idiopathic AVA	Saccular	6.0 × 5.0	CXR, CT, MRI	None	Surgical resection	[3]
Ko	55	M	Arteriovenous fifistula infection	Idiopathic AVA	Saccular	3.7 × 3.0	CXR, CT	End-stage renal disease	Surgical resection	[3]
Ko	66	F	Dyspnea, pulmonary thromboembolism	Idiopathic AVA	Saccular	2.8 × 2.4	CXR, CT	After breast cancer operation	Surgical resection	[3]
Ko	77	F	Trauma	Idiopathic AVA	Fusiform	5.5 × 3.0	CXR, CT	Diabetes mellitus	Conservative	[3]
Ko	56	M	Trauma	Idiopathic AVA	Fusiform	3.2 × 2.7	CXR, CT	None	Conservative	[3]
Ko	46	F	Hemoptysis, lung metastasis	Idiopathic AVA	Fusiform	2.7 × 2.2	CXR, CT	After rectal cancer operation	VATS resection, left upper lobectomy	[3]
Ko	67	F	Stroke	Idiopathic AVA	Fusiform	3.0 × 2.7	CXR, CT	None	Conservative	[3]
Ko	37	F	Cough, hemoptysis	Idiopathic AVA	Fusiform	3.5 × 3.0	CXR, CT, MRI	Bronchopulmonary sequestration	VATS resection, right lower lobectomy	[3]
Ko	34	M	Fever, cough	Idiopathic AVA	Fusiform	4.5 × 2.5	CXR, CT, MRI	Drug addict, mediastinal hemangiomatosis	Conservative	[3]
Choo	79	F	Lower leg weakness	Idiopathic AVA	N/A	3.8	CXR, CT, MRI	Vertebral disk disorder	Conservative	[4]
He	41	F	N/A	Idiopathic AVA	N/A	N/A	CXR, CT, MRI, phlebography	N/A	Surgical resection	[5]
Briones-Claudett	86	F	Dysphagia, paresthesia	N/A	N/A	N/A	CXR, CT, Bronchoscopy, three-dimensional reconstruction	Aspergillus fumigatus infection	Conservative	[6]
Irurzun	N/A	N/A	Cough, wheezing, hiccups	Idiopathic AVA	N/A	N/A	N/A	N/A	Transcatheter embolization	[7]
Figueiredo	78	M	Depressed level of consciousness	Acquired AVA	Saccular	1.2	MRI	Portal hypertension	Conservative	[8]
Guo	28	M	Cough, fever	Acquired AVA	Saccular	N/A	CT, three-dimensional reconstruction	After aneurysm resection	N/A	[9]
Ichiki	76	M	None	Idiopathic AVA	N/A	3.5	CXR, CT, MRI	None	VATS resection	[10]
Mohajeri	45	M	Trauma	Idiopathic AVA	Saccular	3.8 × 2.8	CXR, CT, MRI	None	Conservative	[11]
DeMaio	28	F	Chest pain, neurologic deficits, shock	Acquired AVA	Fusiform	N/A	CT, phlebography	A motor vehicle collision	Stent graft implantation	[12]
Weber	15	M	Chest pain	Idiopathic AVA	Saccular	2.6 × 4.0	CXR, CT, MRI, PET	Pulmonary embolus	Transcatheter embolization	[13]
Favelier	78	F	Chest pain	Idiopathic AVA	N/A	6.0 × 5.0	MRI, phlebography	Pulmonary embolus	Stent graft implantation	[14]
Icard	68	M	Chest pain	Idiopathic AVA	Saccular	6.0	CXR, CT	None	Surgical resection	[15]
Tang	42	M	None	Idiopathic AVA	Saccular	4.2 × 6.7 × 4.0	CT, three-dimensional reconstruction	N/A	Surgical resection	[16]
Du	70	F	Chest pain	Idiopathic AVA	Saccular	4.0 × 3.5 × 3.4	CT, phlebography	None	VATS resection	[17]
Du	53	M	None	Idiopathic AVA	Saccular	5.5 × 3.5 × 4.0	CT	None	Conservative	[17]
Du	60	F	Cough	N/A	Saccular	2.1 × 2.0 × 2.0	CT	None	Oral anticoagulation	[17]
Xie	70	F	Chest pain	N/A	Fusiform	4.0 × 3.5 × 3.4	CT	None	VATS resection	[18]
Morton	73	F	Dysphagia, odynophagia, chest tightness	Idiopathic AVA	Saccular	5.5 × 5.0	CT, phlebography	Chronic obstructive pulmonary disease, gastroesophageal reflux disease, diverticulitis, osteochondroma of the right scapula after surgery	Transcatheter coil embolization	[19]
Wang	53	F	Chest tightness, choking	Idiopathic AVA	Saccular	4.2 × 3.7 × 2.6	CT	None	VATS resection	[20]
Choi	0.25	M	Chest tightness	Idiopathic AVA	Saccular	10	CXR, CT, TTE	Pulmonary embolus	Surgical resection	[21]
Takamori	43	N/A	None	Idiopathic AVA	Saccular	4.7 × 3.5 × 2.4	CT	None	VATS resection	[22]
Zhang	36	M	Chest pain, Chest tightness	N/A	Fusiform	5.5 × 4.0 × 4.2	CT	None	Surgical resection	[23]
Gomez	N/A	N/A	N/A	Idiopathic AVA	N/A	N/A	CT, phlebography	N/A	N/A	[24]
Rothman	26	M	Dachycardia, Chest tightness	Idiopathic AVA	Fusiform	5 × 2.5 × 2.5	CXR, CT	N/A	Stent graft implantation	[25]
Hatachi	56	F	Chest pain	Idiopathic AVA	Fusiform	4.3 × 3.6	CT	None	VATS resection	[26]
Kurihara	73	F	None	Idiopathic AVA	N/A	3.5 × 3.0 × 2.5	CXR, CT	None	Surgical resection	[27]
Zhang	42	M	None	Idiopathic AVA	Saccular	5.0	CT	None	Surgical resection	[28]
Ueda	60	F	None	Idiopathic AVA	N/A	2.5	CXR, CT	None	VATS resection	[29]
Lee	38	F	None	Idiopathic AVA	Fusiform	3.0	CXR, CT	None	VATS resection	[30]
Miura	79	F	None	Idiopathic AVA	Fusiform	6.6 × 6.5 × 4.5	CXR, CT, MRI	None	VATS resection	[31]
Guo	49	F	None	N/A	Saccular	5.0 × 4.0 × 3.0	CT	None	Robot-assisted VATS resection	[32]
Hu	23	M	None	Acquired AVA	Saccular	5.4 × 4.5 × 4.0	CT, three-dimensional reconstruction	ArteriovenousAneurysm	VATS resection	[33]
Obeso	74	F	None	N/A	N/A	1.9 × 1.1	CT	Lung cance	VATS resection, right upper lobectomy	[34]
Davis	70	M	Chest pain	Idiopathic AVA	Saccular	3.3 × 2.6	CT, phlebography	Pulmonary embolism	Stent graft implantation	[35]
Suzuki	79	M	Pleural effusions	Acquired AVA	N/A	5.0	CXR, CT	Mitral and tricuspid valve regurgitation	Surgical resection	[36]
Córdoba	68	M	Syncope	Idiopathic AVA	Saccular	3.8	CXR, CT, MRI	Accessory fissure	N/A	[37]
Seo	72	F	Chest discomfort	Idiopathic AVA	N/A	3.5	CXR, MRI, PET	None	N/A	[38]
Imori	62	F	None	Idiopathic AVA	N/A	2.0 × 1.5	CT, EUS	Lung cancer	Conservative	[39]
Yang	75	F	General weakness, neurologic deficits	Idiopathic AVA	N/A	4.1 × 2.5 × 3.0	CXR, CT	Cerebral infarction	Oral anticoagulation	[40]
Ishikura	51	F	None	Idiopathic AVA	N/A	6.0	CXR, CT	None	Surgical resection	[41]
Ichihara	64	M	Hemoptysis	Acquired AVA	N/A	N/A	N/A	Dilated submucosal bronchial vessels	N/A	[42]
Gnanamuthu	73	M	Cough and mild dysphagia	Idiopathic AVA	N/A	5.0	CXR, CT, PET	Chronic obstructive airways disease	Surgical resection	[43]
Nakamura	37	F	Chest pain and palpitations	Idiopathic AVA	Saccular	11.0 × 9.0	CXR, CT	Pulmonary embolus	Embolectomy	[44]
D’Souza	29	F	Dyspnea	Idiopathic AVA	N/A	5.0	CXR, CR, MRI	Ehlers-Danlos syndrome type IV	Stent graft implantation	[45]
Abad	49	F	None	Idiopathic AVA	N/A	3.0 × 3.5 × 1.5	CXR, CT, MRI	None	Surgical resection	[46]
Person	61	F	Lightheadedness, nausea, and leg weakness	Idiopathic AVA	N/A	N/A	CXR, CT	Hypothyroidism	VATS resection	[47]
Dilege	72	F	Chest pain	N/A	N/A	N/A	CXR, CT, MRI	N/A	Surgical resection	[48]
Bobbio	66	F	None	N/A	N/A	N/A	CXR, CT	Esophageal carcinoma	Surgical resection	[49]
Sakaguchi	52	M	None	Idiopathic AVA	Saccular	4.0 × 3.0 × 3.0	CXR, CT, MRI	None	Surgical resection	[50]
Gallego	64	F	Cough, wheezing	Idiopathic AVA	N/A	3.5	CXR, CT, MRI	None	N/A	[51]
Poll	46	F	None	Idiopathic AVA	N/A	8.0	CXR, CT, MRI	Sickle cell anemia	Conservative	[52]
Watanabe	64	F	Cough, fever	Idiopathic AVA	Saccular	3.0	CXR, CT, MRI, TEE	None	Surgical resection	[53]
Mehta	70	F	None	N/A	N/A	N/A	CXR, CT	Rectal carcinoma	N/A	[54]
Lena	70	M	Hematoma involving the pectoralis major	Idiopathic AVA	N/A	3.0 × 3.0	CXR, CT, TEE	Lung cancer, coronary heart disease	N/A	[55]
Kurihara	62	M	N/A	N/A	Saccular	N/A	N/A	N/A	N/A	[56]
Seebauer	54	F	Superior vena cava occlusion syndrome	Idiopathic AVA	Saccular	15.0 × 7.0	CXR, CT, TEE, phlebography	None	Surgical resection	[57]
Ikushima	N/A	N/A	N/A	N/A	N/A	N/A	4D-flow MRI	N/A	VATS resection	[58]
Bhojwani	17	M	Dysphagia, postprandial emesis	Idiopathic AVA	N/A	N/A	CT	Heterotaxy syndrome	Oesophagostomy	[59]
Savu	74	F	Chest pain	N/A	Saccular	3 × 4	CXR, CT, bronchoscopy	Heart failure	Surgical resection	[60]
Zhou	65	M	None	Idiopathic AVA	Saccular	2.6 × 2.0 × 2.7	CT	N/A	VATS resection	[61]
Zhou	71	F	Dizziness	Idiopathic AVA	Saccular	3.8 × 3.2 × 3.9	CT	Hypertension	Conservative	[61]
Zhang	46	F	None	N/A	Saccular	5.0	CT	None	VATS resection	[62]
Sun	52	M	None	N/A	Saccular	4.4 × 3.4	CT	None	VATS resection	[63]
This case	56	M	Dysphagia	Idiopathic AVA	Fusiform	8.1 × 7.5	CT, bronchoscopy, three-dimensional reconstruction	None	VATS resection	

*F* female, *M* male, *N/A* information not available, *AVA* Azygos vein aneurysms, *CT* Computed tomography, *CXR* chest radiography, *MRI* magnetic resonance imaging, *TEE* transesophageal echocardiography, *PET* positron emission tomography, *VATS* video-assisted thoracoscopic surgery

Currently, there are no guidelines on the optimal treatment of AVAs. In a previous report, conservative observation combined with oral anticoagulation was successful in asymptomatic AVA patients. Ko et al. [3] reported that only 2 of 10 patients with idiopathic AVAs underwent surgical resection immediately after diagnosis. Six patients underwent surgery due to thrombosis and tumor enlargement during follow-up. Patients should be re-evaluated regularly, and surgery or interventional therapy should strongly be considered in cases of compression of adjacent structures, appearance of clinical symptoms, thrombus formation in the aneurysm sac in patients with oral anticoagulation, contraindication to oral anticoagulation, pulmonary embolism, and considerable increase in the diameter of the AVA [1, 3, 18, 20]. In 21 of 72 patients, thoracotomy or thoracoscopic-assisted thoracotomy was performed to remove the AVA. With the advancement of endoscopic techniques, 19 of 72 patients were also reported to have AVAs removed by three-port or four-port thoracoscopic surgery, and 1 AVA was resected by robotic-assisted four-port endoscopy [3–63]. Single-port thoracoscopy causes less surgical trauma and pain. With the development of minimally invasive techniques, surgeon are increasingly selecting the single-port thoracoscope instead of the multi-port thoracoscope in thoracic surgery. We finally decided to select single-port thoracotomy AVA resection which had been no reports until this one. Although the single-port thoracoscope has less space to operate and more interference between operating instruments, the surgeon can overcome that through constant practice. The single-port thoracoscope also has its unique advantages. The single-port thoracoscope lens is in the same direction as the operator's vision. So the operation direction is in the same as the vision on the display that making the operation more accurate. However, the multi-port thoracoscope lens forms an angle with the operator's direction of operation. So there is a visual deviation between the direction of operation and the display, which may increase difficulty and the risk of bleeding for vascular operation. During the surgery, we avoided unnecessary clamping of the mass to prevent bleeding. The proximal end of the AVA was blocked first to prevent thrombus migration from causing pulmonary embolism. At the same time, the surgical plan was formulated based on preoperative three-dimensional reconstruction. We predict the location of arterial branches in advance, which further reduces the risks associated with surgery. Finally, the tumor was successfully resected safely under single-port thoracoscopic surgery. We have proved that single-port thoracoscopic surgery is safe and effective for the treatment of AVA.

At the same time, interventional therapy is a relatively new option for AVA. There are case reports of successful implantation of covered stents within the aneurysm [12, 14, 25, 35], transcatheter embolization of the aneurysms [7, 13] and Amplatzer closure occlusion of tumor blood vessels [35]. Although interventional treatment has less trauma than surgery. However, patients should be treated with surgery in case of excessive physiological curvature of the AV anticipates difficulty in passing the interventional guidewire, compression of adjacent structures, appearance of clinical symptoms, thrombus formation in the aneurysm sac in patients, severe pulmonary embolism requiring surgical intervention. We believe that the surgical resection, especially minimally invasive resection, is simpler and safer in patients with AVAs and therefore generally recommend earlier treatment even if there is no thrombus within the AVA [1, 29]. At the same time, embolization cannot prevent thrombus migration or tumor compression symptoms [13]. Moreover, the embolization device may aggravate compression symptoms after thrombosis.

## Conclusion

In conclusion, some patients may have symptoms such as dysphagia and chest tightness due to tumor compression of the esophagus and trachea. Preoperative three-dimensional reconstruction greatly helps surgeons clarify the disease diagnoses, formulate surgical plans, avoid damage to the surrounding vital organs, and reduce intraoperative bleed. We proved that minimally invasive single-port thoracoscopic surgery is also safe and effective for the treatment of AVA.

## Data Availability

Not applicable.

## Abbreviations

AV: Azygos vein
AVA: Azygos vein aneurysm
CT: Computed tomography
MRI: Magnetic resonance imaging
VATS: Video-assisted thoracoscopic surgery
